# Early immune anergy towards recall antigens and mitogens in patients at onset of septic shock

**DOI:** 10.1038/s41598-018-19976-w

**Published:** 2018-01-29

**Authors:** M. Feuerecker, L. Sudhoff, B. Crucian, J.-I. Pagel, C. Sams, C. Strewe, A. Guo, G. Schelling, J. Briegel, I. Kaufmann, A. Choukèr

**Affiliations:** 1 Laboratory of Translational Research “Stress and Immunity”, Department of Anaesthesiology, University Hospital, LMU Munich, Marchioninistraße 15, 81377 Munich, Germany; 2 Johnson Space Center (JSC), NASA, 1601 NASA Parkway, Houston, Texas 77058 USA; 3 Department of Anaesthesiology, Hospital Munich-Neuperlach, Oskar-Maria-Graf-Ring 51, 81737 Munich, Germany

## Abstract

The pathology of sepsis is typically characterized by an infection and excessive initial inflammation including a cytokine storm, followed by a state of immune suppression or paralysis. This classical view of a two peak kinetic immune response is currently controversially discussed. This study was a sub-study of the randomized clinical Trial SISPCT registered with www.clinicaltrials.gov (NCT00832039, Registration date: 29/01/2009). Blood samples from 76 patients with severe sepsis and septic shock were incubated for 48 h at 37 °C *in vitro* with bacterial or fungal recall-antigens or specific mitogen antigens within 24 hours of sepsis onset. Recall-antigen stimulation led to a severe dampening of normal cytokine release. This immunologic anergy was similarly observed after mitogen stimulation. Moreover, patients under hydrocortisone therapy or with lowered arterial oxygen tension had further reductions in cytokine levels upon B- and T-cell mitogen stimulation. This investigation reveals an early onset of immunoparalysis during sepsis. This immune incompetence in mounting an adequate response to further infections includes previously sensitized pathogens, as seen with recall-antigens. Also, the immune-suppressive role of hydrocortisone and low PaO_2_ is highlighted. Aside from early broad-spectrum antimicrobial therapy, our findings reinforce the need for maximal immunological support and protection against further infections at the onset of sepsis.

## Introduction

Sepsis and sepsis-associated disease states are not only an observation in modern medicine, there have long been many reports dealing with this particular condition in the past. Sir William Osler (1849–1919) observed that patients apparently died from the body’s response to the infection rather than from the infection itself^1^. His observations of the immune system still hold true today, making sepsis a particularly dangerous and tenacious disease. At the end of the 20th century, incidence of sepsis has increased annually by 8.7% in the US, peaking in the year 2000 at 240.4 per 100,000 inhabitants. Although the in-hospital mortality decreased from 27.8% to 17.9%, the total number of deaths continued to rise due to the increase in incidence^2^. Despite all advances in modern medicine and antimicrobial therapy, sepsis and in particular septic shock are still the leading causes for death in critically ill patients in the United States^1,3^. Considering sepsis to cause a comparable number of annual deaths as acute myocardial infarction^4^, further research leading to new therapeutic strategies is directly needed.

Besides systemic inflammatory response syndrome (SIRS), which still is the hallmark sign of sepsis, another contrary condition in the progression of sepsis is described in the literature. It is associated with an inhibition of the immune system, resulting in a lack of response to pathogens, and goes by different names, such as compensatory anti-inflammatory response syndrome (CARS) or immune paralysis^5–7^. This condition causes a severe susceptibility to secondary infection and might be responsible for a significant number of deaths in the later phases of sepsis. There are different hypotheses concerning this immunosuppressive state. It is assumed that a shift from a TH_1_-dominated initial immune response resulting in excessive inflammation and, subsequently, SIRS, to a TH_2_-dominated anti-inflammatory state might contribute to the development of CARS^3^. Different works state that extensive lymphocyte apoptosis during sepsis progression seems to be, at least in part, responsible for the genesis of CARS^8^. Recent findings suggest that hyperinflammation and hypoinflammation are two concurrently developing processes in sepsis, terming it as mixed anti-inflammatory response syndrome (MARS)^6,9^. There are numerous further theories concerning the pathophysiology of immunosuppression in sepsis, including impaired leukocyte recruitment and decreased cell surface protein expression^10^. After all, the exact pathophysiology of sepsis and the accompanying hyperinflammatory and immunosuppressive states are still poorly understood.

To gain better insight into the early phase of septic shock we have recruited patients for the present clinical trial. The main focus was to evaluate the patients’ initial immune function shortly after onset of severe sepsis or septic shock. This was carried out with a recall-antigen whole blood assay, along with specific innate and adaptive immune cell activation assays. We hypothesized a pre-existing global immunosuppression at this time point. Moreover, this report looks at the capacity of these assays to detect effects of additional early hydrocortisone (HC) treatment as well as the immunosuppressive consequences of hypoxia in this patient cohort.

## Results

### Study design and demographic data

The *immune function study* was conducted from June 2011 to February 2013 as part of the clinical trial SISPCT^11^ (www.clinicaltrials.gov: NCT00832039, Registration date: 29/01/2009). In total, 76 patients with severe sepsis or septic shock were included. An age- and sex matched healthy volunteer (HV) control group was recruited between September 2012 and December 2012. No significant group differences could be determined based on age, sex, height, body weight and body mass index (BMI) (Table 1).

**Table 1:** Comparison of general patient and healthy volunteers data, including important Intensive Care Unit scores and therapy variables. ^a^Values are mean ± SD; ^b^Mann-Whitney U test, ^c^t-test, ^d^Chi Square test, ^e^Fisher’s exact test; n.a. = not applied.

Patient and general variables	Patient Group	Healthy Volunteers Group	p
Age [yrs]^a^	60.9 ± 16.6	55.5 ± 12.5	0.15^b^
Body weight [kg]^a^	83.9 ± 24.2 (n = 75)	82.6 ± 19.0	0.22^c^
Height [cm]^a^	171.3 ± 9.5 (n = 73)	175.0 ± 8.0	0.94^b^
Body Mass Index^a^	28.6 ± 8.4 (n = 73)	27.0 ± 6.2	0.57^b^
Sex (male/female)	41/35	8/3	0.24^d^ 0.34^e^
Glasgow Coma Scale (GCS)^a^	6.5 ± 5.2 (n = 76)	15	
Acute Physiology And Chronic Health Evaluation II (APACHE II)^a^	27.4 ± 8.4 (n = 74)	Not assessed	n.a.
Simplified Acute Physiology Score (SAPS II)^a^	65.7 ± 16.2 (n = 74)	Not assessed	n.a.
Ventilation (none/non-invasive/invasive)	13/10/53	11/0/0	n.a.
Vasopressors (yes/no)	70/6	0/11	**4.42e** ^**−10,c**^
Antimicrobial therapy (yes/no)	73/3	0/11	**4.87e** ^**−11,c**^
Hydrocortisone (yes/no)	41/35	0/11	**<0.001** ^**c**^

At the time of enrolment, 96.0% (n = 73) of patients received antimicrobial therapy. In 53% (n = 40) of patients, pneumonia was identified as the primary focus, followed by intra-abdominal foci (19%, n = 14). Pathogen detection was achieved in 51% (n = 39) of the cases. Therein 54% (n = 21) were shown to be gram-negative sepsis, 38% (n = 15) gram-positive and 8% (n = 3) viral. Increased “Simplified Acute Physiology Score II” (SAPS II) and “Acute Physiology and Chronic Health Evaluation II” (APACHE II) scores were seen (Table 1). About 95% (n = 70) of patients were treated with inotropic or vasopressor medications for an average of 14.2 ± 8.3 h (n = 70) at study enrolment. The majority of patients on multiple catecholamines (n = 40) received hydrocortisone within the first 24 h (n = 30). The 90-day mortality was 18.7% (n = 14) with a mean survival of 30.6 ± 33.4 days. Immediately after patient enrolment, blood was drawn for the study. In mean average this was 14.5 hours (SD 5.8 hours) after the first symptoms occurred.

### Blood Samples

#### Complete Blood Count (CBC)

Almost all patients had abnormal CBC results. 11 patients showed leukopenia and 39 patients had leukocytosis (Table 2).

**Table 2:** Complete Blood Count (CBC) and Plasma inflammation marker. Deviation from standard values are marked bold. Data are mean ± SD; n = 51–68.

Septic patients	Standard Value	Mean ± SD	Range
***Complete blood count (CBC)***			
Leukocytes [G/l]	*4.5*–*10.5*	**14.6** ± **10.6 ↑**	0.8–46.8
Erythrocytes [T/l]	*4*.*2*–*5*.*1*	**3.5** ± **0.7 ↓**	2.3–5.5
Hemoglobin [g/dl]	*12*.*0*–*16.0*	**10.7** ± **2.3 ↓**	7.0–18.0
Hematocrit [%]	*36.0–46.0*	**31.3** ± **6.4 ↓**	20.9–50.7
Thrombocytes [G/l]	*150–400*	191.6 ± 97.6	32–571
Mean Corpuscular Volume (MCV) [fl]	*79.0–92.0*	89.8 ± 6.3	67.6–104.0
Mean Corpuscular Hemoglobin (MCH) [pg]	*26*.*5*–*32.5*	30.1 ± 2.4	21.8–35.6
Mean Corpuscular Hemoglobin Concentration (MCHC) [g/dl]	*32*.*0*–*36.0*	33.6 ± 1.5	29.8–36.7
***Plasma inflammation markers***			
CRP [mg/dl]	≤*0*.5	**20.7** ± **14.6 ↑**	0.3–66.5
Interleukin-6 [pg/ml]	≤*5*.9	**18989.6 ± 71652.2 ↑**	26–499,000
Procalcitonin [ng/ml]	≤*0*.1	**16.9** ± **25.5 ↑**	0.1–105.0

#### Plasma Inflammation Markers

Except for one patient with a normal C-reactive protein (CRP) value and another with normal procalcitonin (PCT), all others had an elevated CRP, interleukin (IL) 6 and PCT (Table 2).

#### In vitro Recall Antigen and Mitogen Stimulation Assays

**Figure 1:**
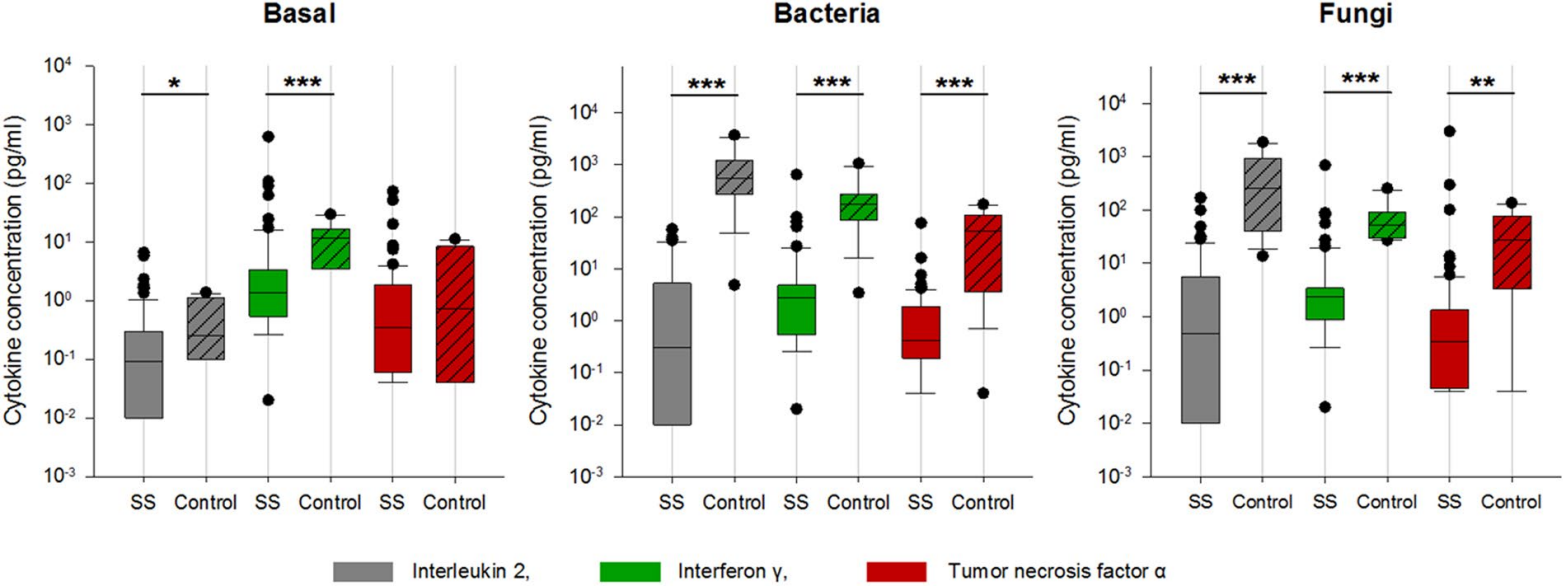
Patients versus control group in unstimulated assay and after stimulation with recall antigens. *SS:* severe sepsis/septic shock patients, *Basal:* unstimulated test assay, *Bacteria:* bacterial antigen mixture, *Fungi:* fungal antigen mixture. Blood samples were taken subsequently to study enrolment (SS) or at a time of subjective physical well-being (control group), respectively. In boxplots, boxes show the median and interquartile range (IQR), whiskers represent the 10th and 90th percentile. Statistically significant differences (Mann-Whitney Rank Sum Test) are indicated as follows: *p < 0.05, **p < 0.01, ***p < 0.001. y-axis: logarithmic scale.

**Figure 2:**
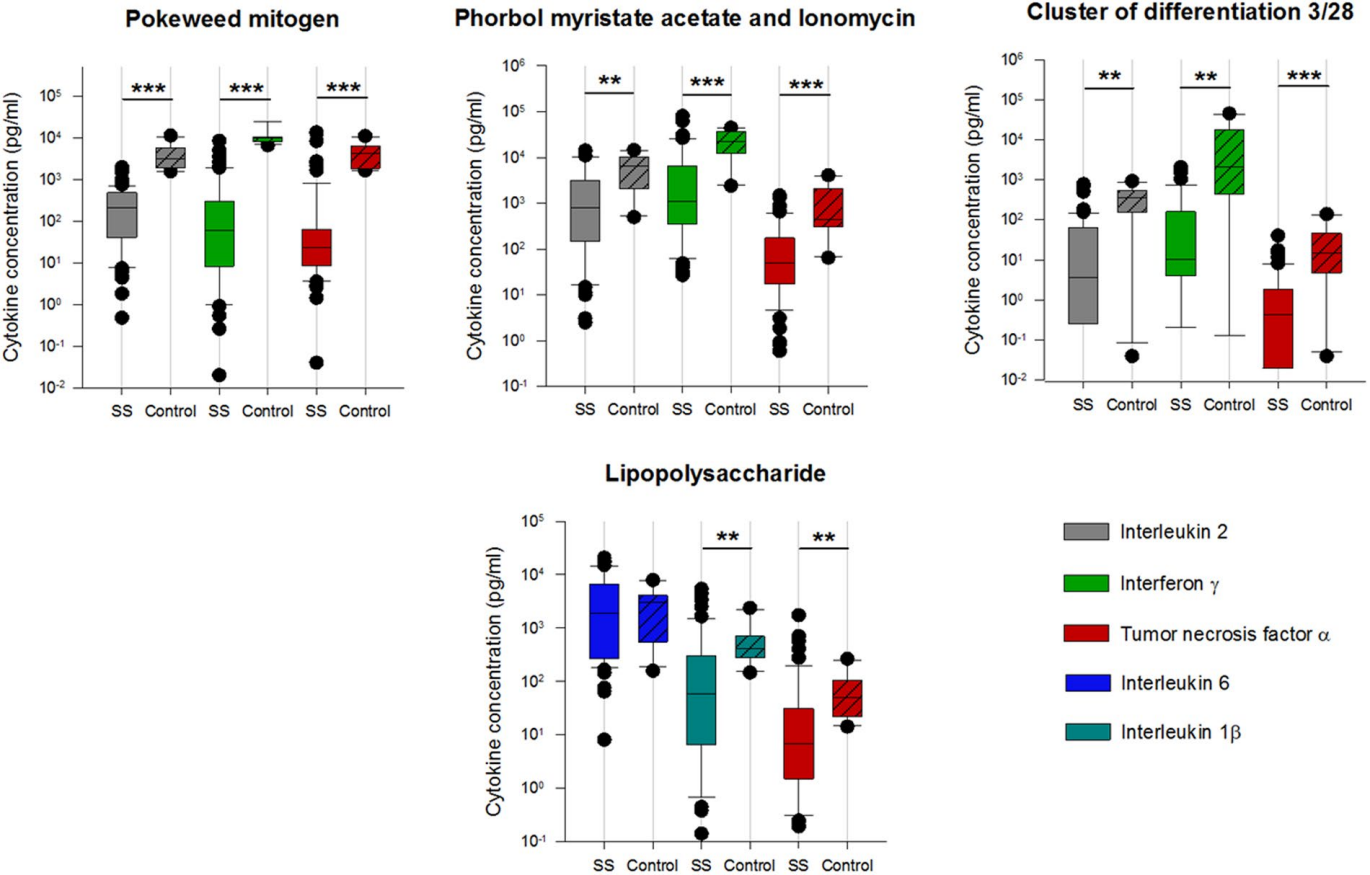
Patients versus control group after stimulation with PWM, PMA, CD3/28 and LPS. PWM: Pokeweed mitogen, LPS: Lipopolysaccharide, PMA-I: Phorbol myristate acetate and Ionomycin, CD3/28: Cluster of Differentiation 3/28, SS: severe sepsis/septic shock patients. Blood samples were taken subsequently to study enrolment (SS) or at a time of subjective physical well-being (control group), respectively. In boxplots, boxes show the median and interquartile range (IQR), whiskers represent the 10th and 90th percentile. Statistically significant differences (Mann-Whitney Rank Sum Test) are indicated as follows: *p < 0.05, **p < 0.01, ***p < 0.001. Y-axis: logarithmic scale.

*Determination of the initial immune function*: Spontaneous cytokine release in the unstimulated (basal) assay showed significantly lower levels of IFN-γ and IL-2 in septic shock/ septic patients (SS). After stimulation with recall antigens, patients had significantly lower pro-inflammatory cytokine levels (interleukin (IL)-2, interferon (IFN)-γ, tumor necrosis-factor (TNF)-α) compared to healthy volunteers (HV), irrespective of the type of antigen used (bacterial, fungal) (Fig. 1). Cell specific stimulation assays with pokeweed mitogen (PWM), lipopolysaccharide (LPS), phorbol 12-myristate 13-acetate (PMA)/Ionomycin, and CD3/CD28 (Fig. 2) also revealed a significant reduction in pro-inflammatory cytokine release (IL-2, IFN-γ, TNF-α) in comparison to HV. Differences between groups after LPS stimulation, which mimics bacterial endotoxins and assesses the innate immune response, were less pronounced but reached statistical significance for Interleukin 1β and TNF-α. Anti-inflammatory interleukins (IL-4, IL-5, IL-10) were significantly lower in sepsis patients and close to the lowest detection limit of the assays. Statistically significant group differences for IL-10 however were only observed after stimulation with soluble antibodies to both CD3 and CD28, given a low significance level (p < 0.05). No differences were observed between male vs. female patients (n.s.).

**Figure 3:**
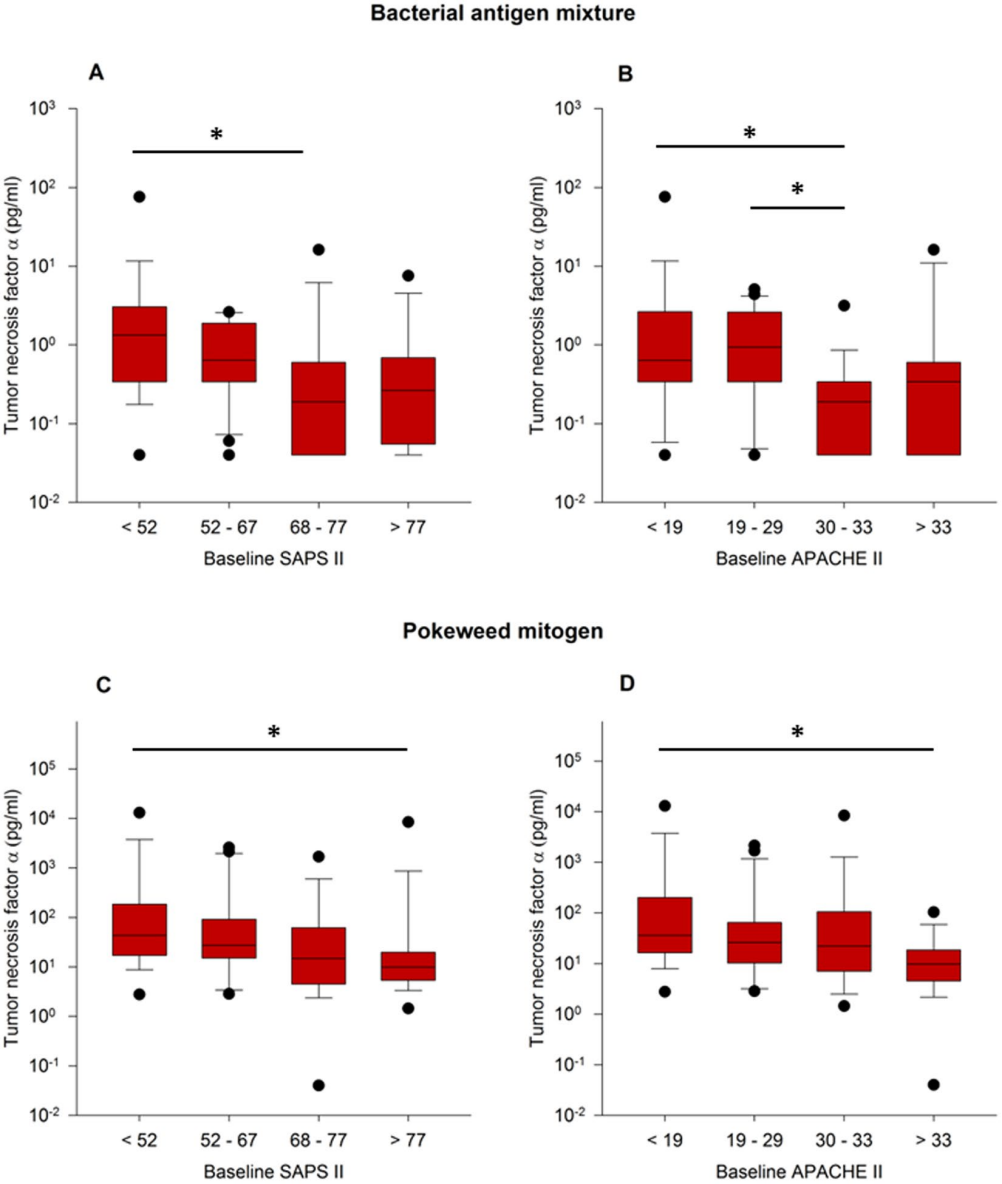
Relationship between disease severity and TNF-α release in Bacteria and PWM assay. TNF-α release from whole blood was correlated with quartile groups for disease severity as measured by SAPS II (**A**,**C**) and APACHE II (**B**,**D**) disease severity classification systems. Panels A and B show hereby stimulations with bacterial antigen mixtures, Panels C and D with Pokeweed mitogen assay. Statistically significant differences (One-way ANOVA on RANKS followed by Dunn’s test) are indicated *p < 0.05.

*Disease severity classification systems and the stimulation assays*: In order to correlate with disease severity, we used SAPS II and APACHE II. Correlation of SAPS II and APACHE II values with the 90-day mortality showed no statistically significant effect although a tendency towards a positive correlation was present (Supplemental Table 1 and Fig. 3).

An increase in disease severity, represented by SAPS II and APACHE II scores, was associated with an impaired TNF-α response both after bacterial antigen (Fig. 3A,B) as well as PWM stimulation (Fig. 3C,D).

**Figure 4:**
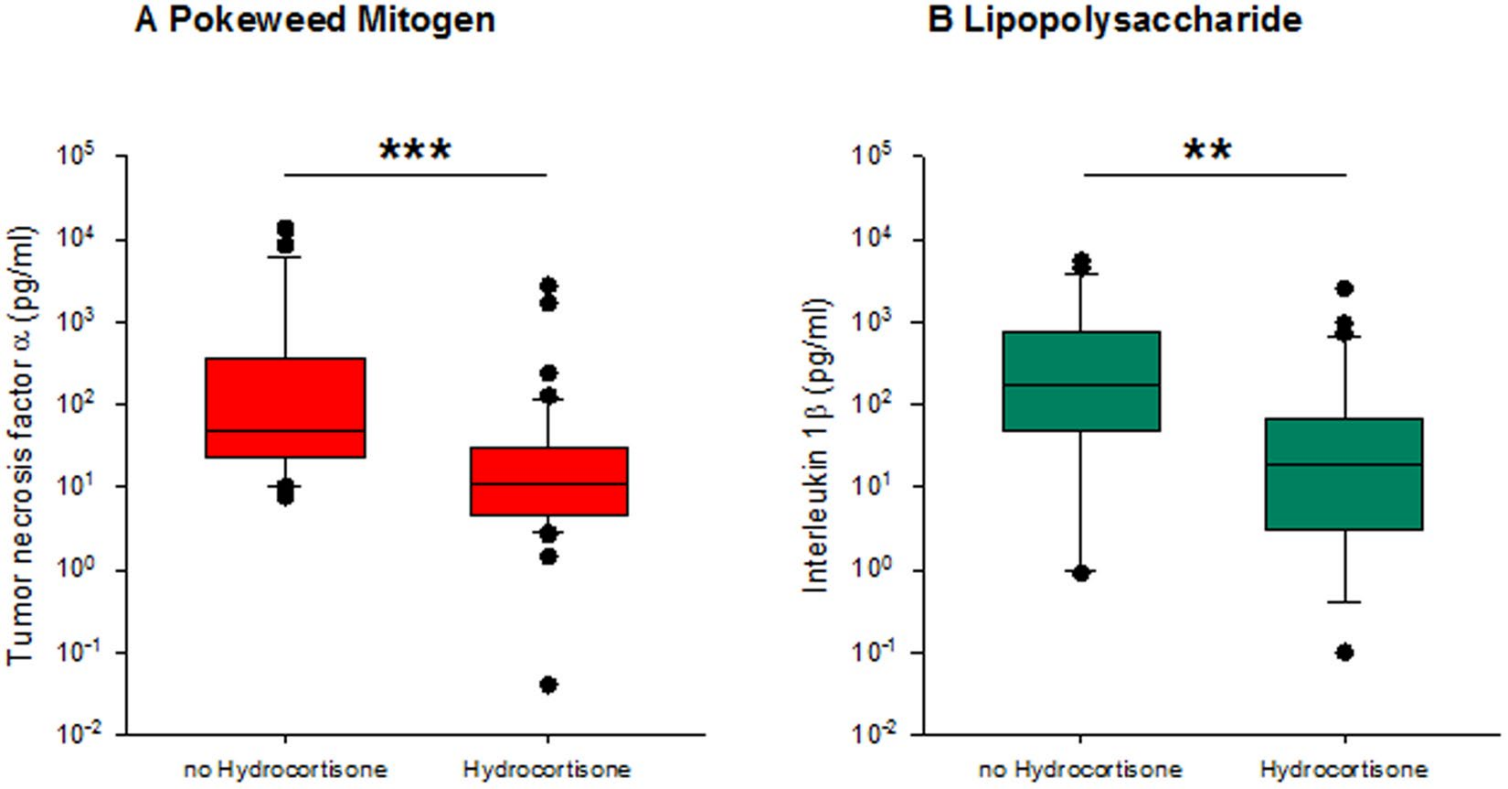
Relationship between hydrocortisone administration and cytokine release. Patients were allocated to two groups (Hydrocortisone (HC) or no HC) for comparison of stimulated cytokine release. (**A**) TNF-α in supernatants of whole blood stimulated with PWM. (**B**) IL-1β in supernatants of LPS stimulated whole blood. Statistically significant differences (Mann-Whitney Rank Sum Test) are indicated as follows: **p < 0.01, ***p < 0.001.

*Immune answers of septic patients with or without hydrocortisone*: In patients with refractory septic shock, a daily dose of 200–300 mg of hydrocortisone is recommended^12^. In our patient collective, 43 patients (56.6%) received hydrocortisone (HC) during the first 24 h of admission to the intensive care unit (ICU). We compared this group to the patients who received no hydrocortisone within this time period with respect to assays cytokine release. Analysis of the two groups revealed no significant differences in the CBC, but the disease severity scores varied significantly (SAPS II: HC: 72.7 ± 14.9, n = 43; no HC: 55.9 ± 12.8, n = 31; APACHE II: HC: 31.0 ± 7.4, n = 43; no HC: 22.4 ± 7.1, n = 31). Patients on HC therapy had higher IL-6 levels compared to the other group (*HC:* 29854 ± 93424 pg/ml, n = 35; *low HC:* 4906 ± 16335 pg/ml, n = 27; Mean ± SD; Mann- Whitney- U test, p = 0.058). Patients receiving hydrocortisone showed a highly significant suppression of TNF-α in the PWM assay (Fig. 4A, p < 0.001) and IL-1β release in the LPS assay (Fig. 4B, p < 0.01). This significant difference was present although the stimulated cytokine responses in all septic patients were enormously reduced as compared to the healthy volunteers.

**Figure 5:**
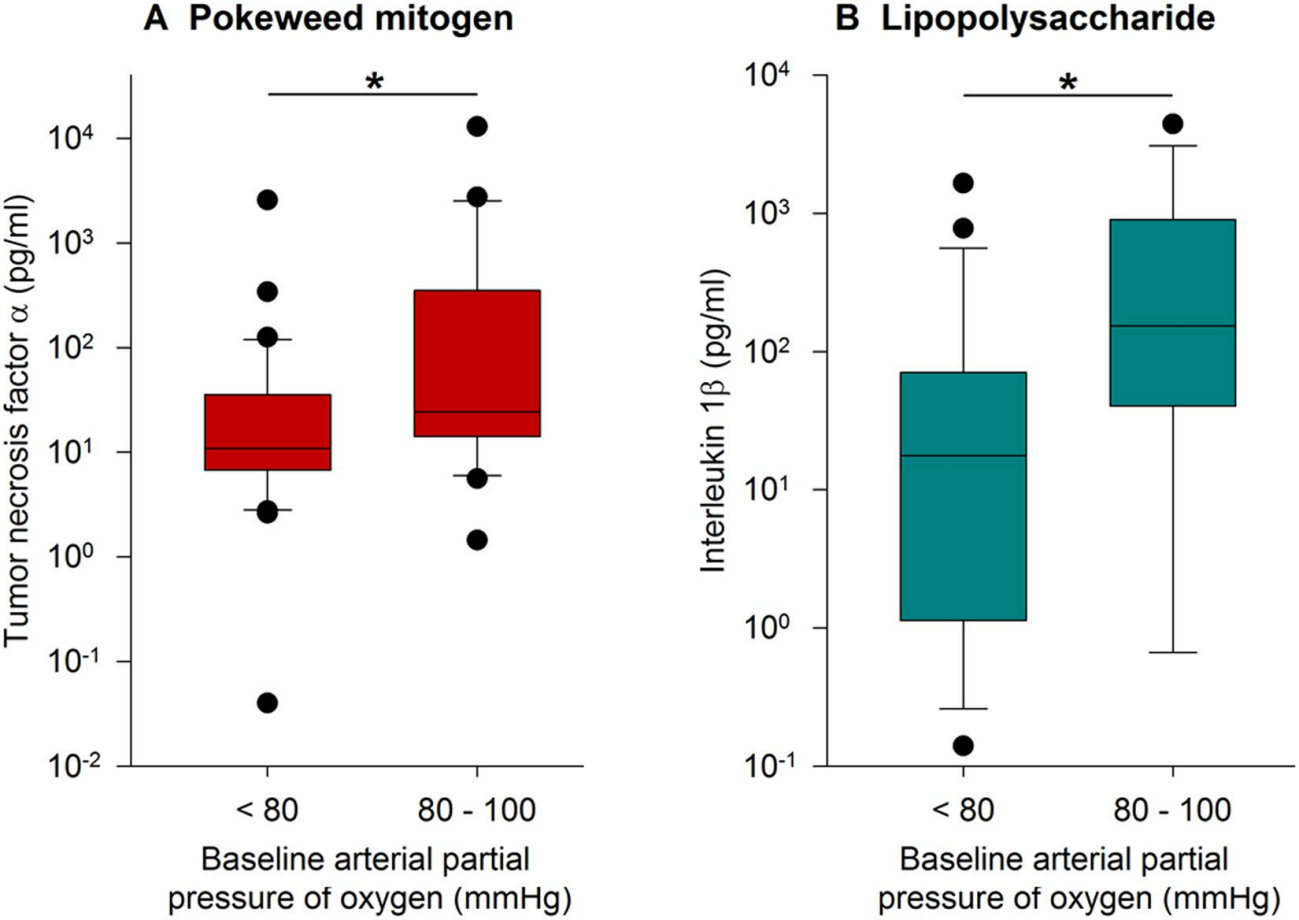
Relationship between PaO_2_ and cytokine release. Patients were allocated to groups regarding their arterial partial pressure of oxygen (*PaO*_2_) for comparison of stimulated cytokine release: hypoxemia (*PaO*_2_ < 80 *mmHg*) and normoxemia (*PaO*_2_ *80–100* *mmHg*). (**A**) TNF-α measured after stimulation with PWM. (**B**) IL-1β measured after stimulation with LPS. Statistically significant differences (Mann-Whitney Rank Sum Test) are indicated *p < 0.05.

*Hypoxemia and immune response*: Using the normal range of PaO_2_ when breathing room air (PaO_2_ 80–100 mmHg^13^), we classified the subgroups as “hypoxemic” patients (PaO_2_ < 80 mmHg) or “normoxemic” patients (PaO_2_ 80–100 mmHg)^14^. Figure 5 shows correlations between PaO_2_ and cytokine release in the assays stimulated with PWM and LPS, respectively. In both assays, supernatant cytokine concentrations (TNF-α, IL-1β) in the “normoxemic” group were higher than those in the hypoxemic group. APACHE II and SAPS II showed statistically similar scores irrespective of the PaO_2_ status.

**Figure 6:**
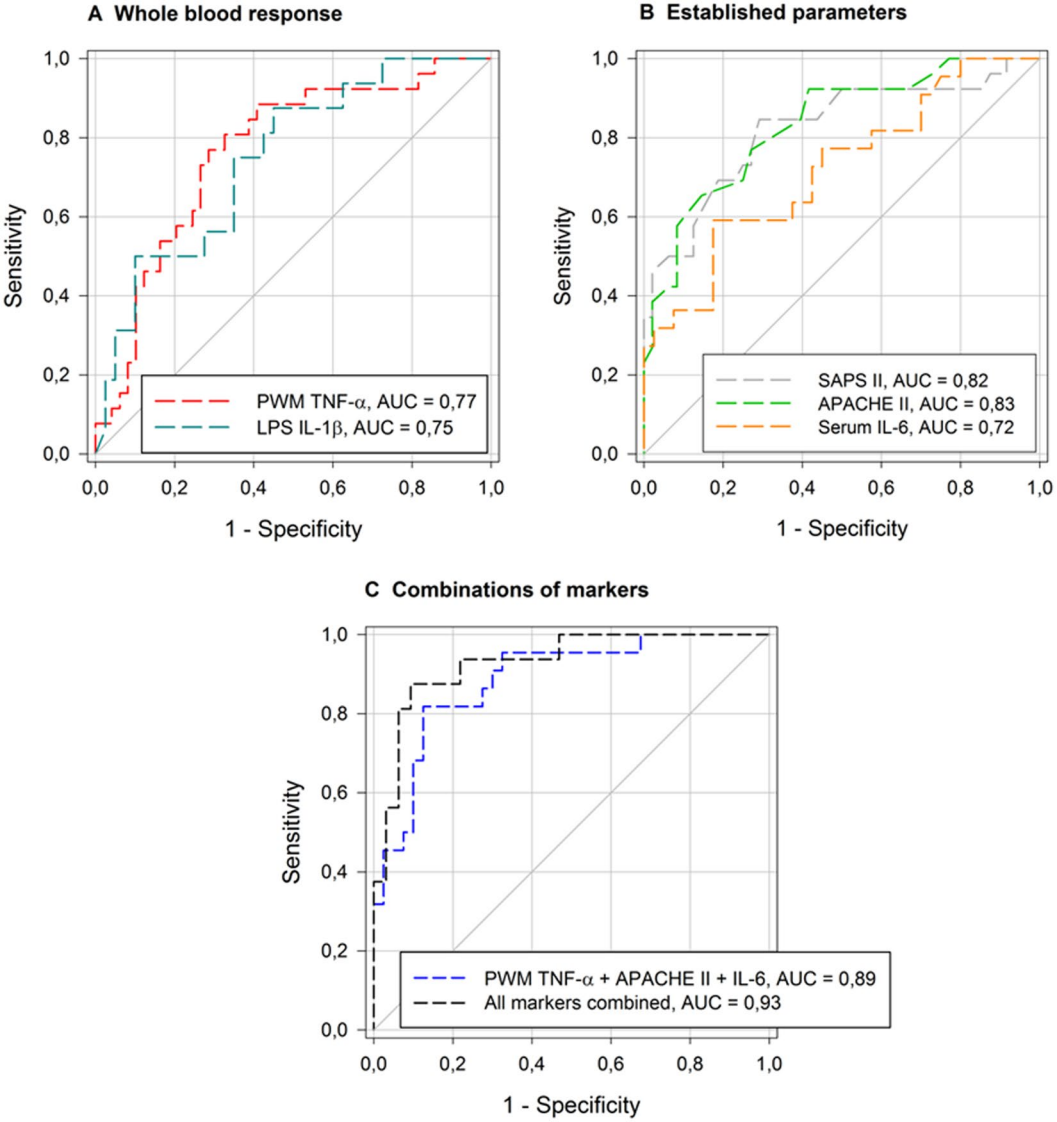
ROC curves for the endpoint‘‘RRT during ICU stay” ROC-curves for (**A**) whole blood response, (**B**) established parameters used in sepsis and **(C**) different combinations of markers referring to (**A** and **B**) regarding the endpoint “initiation of RRT during ICU stay”: *ROC:* Receiver operating characteristic curve, all markers combined: combination of PWM stimulated TNF-α release, LPS stimulated IL-1β release, SAPS II, APACHE II and serum IL-6.

*Receiver operating characteristics Initiation of renal replacement therapy*: For receiver operating characteristic (ROC)-analyses using the endpoint “initiation of renal replacement therapy (RRT) during stay on ICU”, the cytokine assay read-outs from PWM TNF-α and LPS IL-1β as well as disease severity classification systems SAPS II and APACHE II showed similar results, with high statistical significance (Fig. 6).

## Discussion

Testing blood samples from septic patients with a new *in vitro* recall antigen whole blood assay^15^ and immune-cell-specific and unspecific mitogenic stimulation assays revealed, that the human immune system is in a paralyzed state at the onset of sepsis. This blunted immune response from monocyte, T- and B-cell activation can account for the almost entirely extinct cytokine release upon bacterial and fungal recall antigen stimulation. These responses and the immunologic memory to such re-call antigens is one of the key adaptive immune competencies of the host when re-exposed to known pathogens. This old preserved mechanism seems entirely suppressed at the onset of sepsis and might be considered as one explanation for the long lasting immune compromised state and high morbidity.

These findings expand our understanding of the consequences of severe immune dysfunction, frequently described as “immunoparalysis”^16^, by using this battery of *ex vivo* stimulation assays. Furthermore, this clinical trial demonstrates that patients who received stress doses of hydrocortisone (HC) had even more suppressed immune responses. Immune suppression in septic shock patients was further aggravated when the degree of oxygenation resulted in PaO_2_ levels lower than 80 mmHg.

Early on in the development of sepsis, it is classically described that an initial excessive inflammatory state occurs, typically known as a cytokine storm^3^. The entire organism appears to be in an ultimate inflammatory state with pro-inflammatory processes adding up, resulting in tissue injury, organ failure and further inflammation. Pro- and anti-inflammation are two concurrently developing processes emerging early in these pathophysiologic processes^9,17,18^, which are most commonly referred to as mixed antagonist response syndrome (MARS). In the literature, there is relatively little information on the exact kinetics of septic conditions and inflammatory responses. Tamayo *et al*.^17^ described that pro- and anti-inflammatory responses are simultaneously regulated in the first onset of sepsis. Herein, plasma levels of IL-6 and IL-8 were up-regulated but also IL-10. Murine sepsis models based on cecal ligation and puncture (CLP) showed that the systemic inflammatory response represented by elevated levels of pro-inflammatory cytokines in the blood plasma began to emerge within 2–8 hours after the insult, depending on the individual cytokine^19^. Anti-inflammatory processes are assumed to begin within the first 24 hours in human sepsis^20^.

### Overall immune response in sepsis

The here observed ambivalent behavior of high IL-6 serum levels and corresponding low whole blood assay responses can be attributed to the finding that in subacute phases of infectious conditions, the resulting “cytokine storm” is not a physiologic reaction to pathogens, but rather a massive liberation of so-called damage-associated molecular patterns (DAMPs)^21^. The degree of tissue injury and thus the disease severity, as seen by a marked rise in pro-inflammatory markers such as CRP and IL-6, is negatively correlated with the whole blood response to immunologic stimulants. This results in a heightened susceptibility to secondary infections and thus late-phase mortality. The critical players include all immunologic cells and especially cells with leukocytic origin. Regardless of leukocyte differentiation, the cell count in itself already indicates an upset in the immune response. Unsurprisingly, our patients with moderate leukocytosis had the best immune response, whereas both leukopenia as well as severe leukocytosis were associated with increased immune dysfunction. This suggests that higher disease severity is related to the lowest whole blood assay responses.

Furthermore, when correlating assay response in the latter patient group with SAPS II and APACHE II, this relation between significantly impaired immune function with disease severity scores was demonstrated again. Both scores incorporate physiologic parameters which are typically deranged in cases of organ failure and tissue injury^22,23^. These results also substantiate the aforementioned relationship to inflammatory parameters, that our assays were able to help further quantifying disease severity from an immunologic standpoint.

### Pan-immune cell paralysis

The battery of assays display a lack of adequate immune responsiveness in almost all major leukocyte lineage cells, stimuli and antigens used. Recall antigen responses to bacterial toxoids and fungal antigens as well as to polyclonal B- and T-cell activation were highly suppressed and almost entirely blunted. Selective activation of T-cells via co-receptor CD3 and the co-stimulant CD28 was also strongly suppressed in septic patients. In light of the mechanisms of Ca^++^-ionophore (Ionomcyin)/PMA action which leads to reduced T cell activation and cytokine synthesis, a reduction in the signaling capacity of the proteinkinase C (PKC) pathway can be anticipated. Moreover, activation of CD14/TLR4 from innate immune cells (monocytes and granulocytes^24^) through the endotoxin stimulus LPS led to a strong and significant reduction in the release of several key cytokines (IL-1β, TNF-α). This activation may be linked to the PKC pathway and its isoforms^25^ as a key cellular target potentially explaining this cell-mediated immunologic anergy. The role of PKC isoforms in separated cell subsets could not be further elucidated using complementary assays due to the limited blood volumes available.

The highly suppressed cytokine responses to the strong mitogenic (PWM) and receptor specific stimuli (LPS) were still in a well detectable range which allowed for comparisons of either activation or further suppression in sub-cohorts of patients. PWM and LPS assay results were identified as suitable and included in further analyses for the role of HC, oxygenation (Figs 4 and 5) and other Receiver Operating Characteristic (ROC) findings (see supplemental Table 1 and supplemental Fig. 3).

### Effects of hydrocortisone and oxygenation

A highly significant suppression of pro-inflammatory cytokine release was observed in patients receiving stress doses of HC. Considering the clinical indications as well as effects of HC therapy, it is in many ways a double-edged sword. The genomic effects of HC results in immunosuppression which impairs the whole blood assay response and further dampens the immune cells performance in raising a TH1 response. One must take into account that according to clinical guidelines, application of HC should be restricted to patients in refractory septic shock, where hemodynamic stability cannot be restored despite adequate fluid resuscitation and vasopressor use^12,26^. Within our patient population, this applies to patients in the most fulminant phase of septic shock and defines a high severity of the septic state. Therefore, the observed immune function differences between the two sub-groups (low and high HC) could be also due to patient selection as indicated in the administration of hydrocortisone in septic shock.

Hypoxemia can impact the immune response, as shown by our and other studies^27^. Hypoxia-induced anti-inflammatory mechanisms are mediated by the A2 adenosine receptor (A2AR)^28^, HIF and other pathways^29,30^. The anti-inflammatory effects are enabled by various mechanisms such as the inhibition of oxidative burst and a reduction in platelet activation minimizing microvascular occlusion; especially through a reduction in pro-inflammatory cytokines being released (mainly TNF-α or IFN-γ) and an increased release of anti-inflammatory cytokines such as IL-10 or IL-4, suggesting a shift of the lymphocyte TH1/TH2-equilibrium towards the TH2-pathway^31,32^. In the here presented study, we observed a greater immune suppressive response in the hypoxemic patient population. The disease scores and elevated levels of serum lactate as a surrogate parameter for hypoxia were used to identify the state of hypoxia. The blood assays show a significant attenuation of the TH1-responses (IL-1β, TNF-α) when PaO_2_ was below 80 mmHg as compared to normoxemic conditions. The TH2 responses were almost uniformly low and close to the detectable threshold of the respective cytokine assays (e.g. IL-10, PMA stimulation, PaO_2_ < 80 mmHg: 11.4 ± 16.0 pg/ml), suggesting that a TH2 shift and the role of HC or oxygen play a more significant role than the dampening of TH1 responses.

### Role of the immune response in the prediction of renal replacement therapy (RRT)

Acute renal failure (ARF) is a common complication of severe sepsis or septic shock. It is not only an indicator of disease severity, but also an independent risk factor for death, occurring in as often as 41% severe sepsis and septic shock cases in a 2007 prevalence study carried out in German ICUs^33^. ARF and the initiation of renal replacement therapy are predictors of unfavorable disease progression and mortality. Sepsis survivors are at high risk for suffering from chronic kidney disease^34^. While considering the endpoint “initiation of renal replacement therapy”, the immune dysfunction of RRT patients became apparent. ROC analyses using TNF-α and IL-1ß from the *in vitro* assays after stimulation with PWM and LPS can sensitively and specifically predict need for RRT, as good as the widely used SAPS II, APACHE II scores. Combining these immune functional parameters with Serum IL-6, SAPS II and APACHE II, one could generate a useful predictive value in the ROC for RRT with high specificity and sensitivity. The predictive value exceeds that of the conventional parameters in use today. These findings show that immune responses to strong immunologic stimuli (PWM, LPS) might be an additional immune functional tool which can help predicting the need for RRT when combined with established markers and scoring systems in sepsis patients. Parameters on its own, even when a strong positive correlation can be established, are not sufficiently powerful predictors and should be aggregated for better utility. This could be used clinically to identify patients at high risk for receiving RRT and to adjust treatment strategies early.

### Consequences

#### Hygiene, reverse isolation and quarantine

Our data demonstrate that in the very first moments after septic shock onset, immune competence is severely compromised and adaptive immune system responses seem almost non-existent. Liu *et al*.^35^ were recently able to show in 35,000 sepsis patients that with every hour of delay in antibiotic treatment within the recommended 6 hour window, mortality increases. Patients suffering from critical illness and associated immune suppression are also easy targets for secondary infections from viruses and opportunistic pathogens^36,37^. More direct anti-infective therapy does not seem effective when dealing with this issue in sepsis. As soon as one organism is successfully eliminated with antibiotics, antimycotic or virostatic agents, another one not covered by the first round of medications or by the immune system often leads to a superinfection^36^, and many of these pathogens tend to be multidrug-resistant^37^.

In order to minimize the risk of acquiring an opportunistic infection, patients benefit from strict compliance with infection prevention and control^38^. More efforts should be invested in creating better awareness for this issue amongst medical personal. Moreover, temporary reverse isolation and quarantine, typical in other immune suppressed patients i.e. organ transplantation post-op, could have beneficial effects. Although a recent review showed that noncompliance resulted in a paradoxical increase in adverse events^38^.

#### Restriction of hydrocortisone administration

Administration of hydrocortisone in sepsis should be considered on a case by case basis, since HC can further weaken the immune response. The effects of both high-dose as well as low-dose corticosteroids were examined previously in septic patients, none of which showed conclusive improvement in survival^39,40^. There is evidence that an earlier reversal of shock may be accomplished by corticosteroid application^40,41^. As such, corticosteroid administration in current guidelines is restricted to cases of refractory septic shock that does not respond adequately to fluid resuscitation and vasopressor therapy^12,26^. Our data on the additional immunosuppressive effects is in line with current recommendations that the use of corticosteroids in sepsis should be limited to refractory cases.

#### Immune function monitoring and modulation

Severe immunosuppression at the onset of sepsis as seen in this trial and increasingly being discussed in recent literature suggest the potential role of immune modulatory therapies such as GM-CSF or IFN-γ^42^. One of the latest reviews on this topic concluded that in the absence of deleterious side effects from GM-CSF administration in sepsis patients, multiple clinical benefits such as rapid recovery from infection, reduced length of hospital stay and decreased need for mechanical ventilation were seen^43^. The immune cell expansion and other potential immune stimulatory therapies in sepsis are compelling concepts. With increasing evidence that patient deaths are a consequence of the immunosuppressive state, immune enhancing drugs could become the next milestone in sepsis therapy^16^.

## Conclusion

The stimuli used in these whole blood immune assays are based on the principles of re-call antigen responses, specific T and B cell answers or innate cell activation. Taken together it reflects the body’s ability to mount an immune response against a broad spectrum of pathogens, showing anergy and immune-paralysis early on in sepsis. This suppressed state of the immune system requires immediate additional protection against opportunistic infections including an early anti-infective treatment, proper hygiene measures and reverse isolation or quarantine. The roles of HC therapy and PaO_2_ values should also be considered. The broadly used early hydrocortisone application in septic shock should be carefully evaluated on a case by case basis. This study reveals that further research needs to be done to establish a profound marker and immune functional assay – directed therapy.

## Limitations and Considerations

The focus of this investigation was the clinical evaluation of the immune competence upon further distinct immune stimulation in whole blood. Due to recruitment time point and the overall clinical study setup (including strict blood volume limitations) it was not possible to test for specific cell subsets, cell numbers or cell viability under all conditions. To overcome some concerns, *in vitro* experiments were carried out to test for the effects of the assay incubation on the apoptosis and necrosis of the cytokine producing cells. Here CD3, CD4, CD8 and NK cells as well as granulocytes revealed only a moderately increased immune cell apoptosis and necrosis after 48 h assay incubations (e.g. range of necrosis was ~1% in non-stimulated T-cells and up to 10% in PWM stimulated assays).

Moreover, data as presented on this clinical study were analyzed and presented as they were collected, and no extreme outlier exclusion or other statistical tools were applied in order to evaluate the strength and weaknesses of this immune assay approach test to function despite the huge inter-individual pattern. The authors are aware that patients with severe sepsis are a very heterogeneous group and preexisting co-morbidity impacts the longterm outcome as Mansur *et al*. described^44^. As our study focused primarily on the onset of sepsis, co-morbidities were not taken into calculations.

Furthermore, the here presented control group is small but data of the controls are in the margins of what other control experiments in healthy male and female have shown. This presented control group was run in the study period, with the same operators, assay procedures (incl. reagent’s lot etc.) and subsequent analyses.

Samples from healthy volunteers were taken venously and from an arterial line in the septic patients which may have led to some bias. Any kind of inflammatory or systemic diseases were excluded in the healthy subjects and no relevant difference between arterial and venous blood shall be hence anticipated. Moreover, in the light of this knowledge and due to the very strict handling of ethical boards to minimize risks to volunteers, an arterial draw was not possible in the healthy subjects as it seemed hardly justifiable due to an increased risk of potential severe side effects such as malperfusion or necrosis. The blood draw from an arterial line was performed in septic patients to avoid any contamination or bias (dilution) as if blood would have been taken from central i.v. line, which is used otherwise for infusion therapy or drug administration or parenteral nutrition. Interestingly, several publications also addressed the question e.g. if proteins or functions of blood components change in arterial or venous blood. Kelly *et al*.^45^ were able to show that in patients with COPD, biomarkers were comparable in arterial and venous blood samples. Some other reports^46,47^ describe in severely sick patients some differences indicating some advantage to the draw of blood from the arterial line in inflammatory lung disease, but however still, these reports overall judge a similar value of venous to arterial blood. Fernández-Serrano *et al*.^46^ report some preference to arterial blood when lung sick people are investigated. In the current study on sepsis the lung was often affected and one might anticipate-since we have drawn arterial blood-that the “better sample” was collected in the study population. Overall a bias in the immune parameters analyzed just because of the different sites of blood draw in healthy volunteers or septic patients, respectively, can be excluded to a large extent.

## Methods

### Study design

The present *immune function study* was part of a prospective, randomized, multicenter clinical trial named “Placebo Controlled Trial of Sodium Selenite and Procalcitonin Guided Antimicrobial Therapy in Severe Sepsis” (SISPCT, NCT00832039)^11,48^.

#### Informed Consent

Ethical study approval for additional experiments performed at our center was obtained as a local amendment to the approved SISPCT study [Eudra-CT-Nr. 2007-004333-42] from the ethical board of the University of Jena.

After positive patient screening, written informed consent signed by the patient or by the legal representative had to be present for enrolment. In case of withdrawal, the patient was immediately excluded from the study and no further follow-up was performed.

All reported experiments and methods were approved by the ethical board of the University of Jena and were in accordance with the relevant guidelines and regulations. The respective experiment protocols were also approved by the ethical committee of the University of Jena.

### Data recording

In addition to the acquisition of data on demographics, biometrics, past medical history and clinical and laboratory findings, the disease severity classification scores Simplified Acute Physiology Score II (SAPS II)^22^ and Acute Physiology And Chronic Health Evaluation II (APACHE II)^23^ were calculated. Furthermore, the Glasgow Coma Scale (GCS) was assessed. The control group was small but based on our previous experiences with other healthy volunteers it was in the margins of what other control experiments in healthy male and female have shown and it was hence considered a valid size, also since it was matched to the expected age and gender composition of the studied patients at the time of the study. In the volunteers, we have been also using the same lot of reagents/antigens and the exactly same methods and operators to try minimizing the risk of a methodologic bias. All recorded data and analyses were anonymized and stored in a database (SPSS® Statistics 21, IBM Corp., New York City, NY, USA).

### Study specific blood Sample Collection

Immediately after patient enrolment, 9 ml blood was collected from an *in situ* arterial line in lithium-heparinized tubes (Sarstedt, Nümbrecht, Germany). In the control group, blood was collected by venipuncture of a cubital vein.

#### Blood Processing

*Complete Blood Count* (*CBC*): Erythrocyte, leukocyte and platelet count as well as hemoglobin, hematocrit, mean corpuscular volume (MCV), mean corpuscular hemoglobin (MCH) and mean corpuscular hemoglobin concentration (MCHC) were assessed upon admission as standard at the intensive care unit (ICU) (Institute of Laboratory Medicine, University of Munich, Germany).

#### Plasma Inflammation Markers

C-reactive protein (CRP), interleukin-6 and procalcitonin (PCT) were routinely assessed upon ICU admission and measured according to standard procedures (Institute of Laboratory Medicine, University of Munich, Germany).

#### In vitro Recall Antigen and Mitogen Stimulation Assays

Immediately following sample collection, 400 µl of whole blood were transferred into assay tubes prefilled with DMEM (Dulbecco´s Modified Eagle’s Medium Nutrient Mixture F-12 HAM, Sigma-Aldrich, Steinheim, Germany) and the different stimulants, as previously described^15^. The *in vitro* recall antigen and mitogen stimulation assay tubes contained DMEM only or DMEM and either a bacterial recall antigen mixture containing Diphteria-, Tetanus- and Pertussis-toxoid or a fungal antigen mixture containing Candida-Lysate and Trichophyton-Lysate. Additionally, the following mitogens were used: 1) Pokeweed mitogen (PWM), a strong immune activator, induces mitosis in T and B lymphocytes in a non-receptor specific fashion^49,50^; 2) Phorbol-12-myristate 13-acetate (PMA/Ionomycin), an unspecific activator of Protein kinase C (PKC)^51^ affecting multiple cell types^52^ but is also reported as a pan-specific activator of B-cells^53^ and mitogen for T lymphocytes^54^; 3) CD3/28 mixture, which activate T cells via the T cell receptor (CD3) and the cell receptor (CD28), CD28 provides, via binding to antigen presenting cell, costimulation for T cell activation^55^; 4) lipopolysaccharide (LPS), targets the innate pathways via CD14 cell surface receptors and Toll like receptor (TLR) 4 signaling cascades^56^.

Incubation time was 48 h at 37 °C. The supernatant was subsequently transferred into Eppendorf tubes and immediately frozen at −80 °C for future cytokine analyses. Frozen supernatants were measured after thawing in a blinded fashion by Luminex xMAP® technology (Bioplex®) with commercially available reagents from BioRad-Laboratories Inc. (California, USA) according to the manufacturer´s guidelines. The concentrations of the pro- and anti-inflammatory cytokines were analyzed (pg/ml).

### Statistical analyses

In order not to distort raw data, but to fully illustrate the variability of extreme responses and to investigate the effects under real clinical conditions, no outlier analysis was performed and all data in this study cohort was kept for analysis. After testing for normal distribution, data were analyzed either by Student’s T-test, Mann-Whitney Rank Sum Test or One-way analysis of variance (ANOVA) on ranks followed by Dunn´s post-hoc test. Correlation analyses were performed using Pearson’s correlation coefficient or Spearman rank-order correlation coefficient, dependent on presence of a normal distribution. All p-values were calculated in a two-sided manner and statistical significance was set at a p-value of 0.05.

For better comparability of non-normally distributed data, variable values were divided into specific groups (indicated in the individual charts) or four quartile groups, with the 25th, 50th and 75th percentile representing the cut-off values for group allocation.

The predictive value of the *in vitro* cytokine release of TNF-α, IFN-γ and IL-2 after PWM challenge in patients receiving extracorporeal *r*enal *r*eplacement *t*herapy (RRT, dialysis) while in the ICU was further assessed alone or in combination with other markers of disease severity under a receiver operating characteristic (ROC) curve. ROC curves were applied to obtain cut-off values for sensitivity and specificity for the respective cytokines as well as the need for dialysis.

Results are expressed as means ± SD in tables and as boxplots in graphs. Boxes show the median and interquartile range (IQR), whiskers represent the 10th and 90th percentile. Data are plotted and were statistically analyzed using IBM SPSS® Statistics 21 as well as SigmaPlot 11.0 (Systat Software Inc., San Jose, California, USA).

## Electronic supplementary material

Supplemental material

## References

[1] Martin GS (2012). Sepsis, severe sepsis and septic shock: changes in incidence, pathogens and outcomes. Expert Rev. Anti. Infect. Ther..

[2] Martin GS, Mannino DM, Eaton S, Moss M (2003). The epidemiology of sepsis in the United States from 1979 through 2000. N. Engl. J. Med..

[3] Hotchkiss RS, Karl IE (2003). The pathophysiology and treatment of sepsis. N. Engl. J. Med..

[4] Angus DC (2001). Epidemiology of severe sepsis in the United States: analysis of incidence, outcome, and associated costs of care. Crit. Care Med..

[5] Rittirsch D, Flierl MA, Ward PA (2008). Harmful molecular mechanisms in sepsis. Nat. Rev. Immunol..

[6] Wiersinga WJ (2011). Current insights in sepsis: from pathogenesis to new treatment targets. Curr. Opin. Crit. Care.

[7] Ertel W (1997). Inhibition of the defense system stimulating interleukin-12 interferon-gamma pathway during critical Illness. Blood.

[8] Hotchkiss RS (1999). Apoptotic cell death in patients with sepsis, shock, and multiple organ dysfunction. Crit. Care Med..

[9] Osuchowski MF, Craciun F, Weixelbaumer KM, Duffy ER, Remick DG (2012). Sepsis chronically in MARS: systemic cytokine responses are always mixed regardless of the outcome, magnitude, or phase of sepsis. J. Immunol..

[10] Huttunen R, Aittoniemi J (2011). New concepts in the pathogenesis, diagnosis and treatment of bacteremia and sepsis. J. Infect..

[11] Bloos F (2016). Effect of Sodium Selenite Administration and Procalcitonin-Guided Therapy on Mortality in Patients With Severe Sepsis or Septic Shock: A Randomized Clinical Trial. JAMA Intern. Med..

[12] Reinhart, K. *et al*. Prevention, diagnosis, therapy and follow-up care of sepsis: 1st revision of S-2k guidelines of the German Sepsis Society (Deutsche Sepsis-Gesellschaft e.V. (DSG)) and the German Interdisciplinary Association of Intensive Care andEmergency Medicine (Deutsche Interdisziplinare Vereinigung fur Intensiv- und Notfallmedizin (DIVI)). G*er*. *Med*. *Sci*.*: GMS e-journal* 8, Doc14, 10.3205/000103 (2010). 10.3205/000103 PMC2899863 20628653

[13] Jacob, S. & Cory, F. In *Common Surgical Diseases* (eds J. A. Myers, K. W. Millikan, & T. J. Saclarides) Ch. 97, 391–394 (Springer New York, 2008).

[14] respiratoryupdate. *Levels of Hypoxemia*, http://www.respiratoryupdate.com/members/Levels_of_Hypoxemia.cfm (2015).

[15] Feuerecker M (2013). A corticoid-sensitive cytokine release assay for monitoring stress-mediated immune modulation. Clin. Exp. Immunol..

[16] Leentjens J, Kox M, van der Hoeven JG, Netea MG, Pickkers P (2013). Immunotherapy for the adjunctive treatment of sepsis: from immunosuppression to immunostimulation. Time for a paradigm change? Am. J. Respir. Crit. Care Med..

[17] Tamayo E (2011). Pro- and anti-inflammatory responses are regulated simultaneously from the first moments of septic shock. Eur. Cytokine Netw..

[18] Novotny AR (2012). Mixed antagonist response and sepsis severity-dependent dysbalance of pro- and anti-inflammatory responses at the onset of postoperative sepsis. Immunobiology.

[19] Remick DG, Newcomb DE, Bolgos GL, Call DR (2000). Comparison of the mortality and inflammatory response of two models of sepsis: lipopolysaccharide vs. cecal ligation and puncture. Shock.

[20] Frazier, W. J. & Hall, M. W. Immunoparalysis and adverse outcomes from critical illness. *Pediatr*. *Clin*. *North Am*. **55**, 647–668, xi, 10.1016/j.pcl.2008.02.009 (2008). 10.1016/j.pcl.2008.02.009 PMC2474674 18501759

[21] Zhang Q (2010). Circulating mitochondrial DAMPs cause inflammatory responses to injury. Nature.

[22] Le Gall JR, Lemeshow S, Saulnier F (1993). A new Simplified Acute Physiology Score (SAPS II) based on a European/North American multicenter study. JAMA.

[23] Knaus WA, Draper EA, Wagner DP, Zimmerman JE (1985). APACHE II: a severity of disease classification system. Crit. Care Med..

[24] Haziot A, Tsuberi BZ, Goyert SM (1993). Neutrophil CD14: biochemical properties and role in the secretion of tumor necrosis factor-alpha in response to lipopolysaccharide. J. Immunol..

[25] McGettrick AF (2006). Trif-related adapter molecule is phosphorylated by PKC{epsilon} during Toll-like receptor 4 signaling. Proc. Natl. Acad. Sci. USA.

[26] Dellinger RP (2013). Surviving sepsis campaign: international guidelines for management of severe sepsis and septic shock: 2012. Crit Care Med..

[27] Chouker A (2008). Critical role of hypoxia and A2A adenosine receptors in liver tissue-protecting physiological anti-inflammatory pathway. Mol. Med..

[28] Kaufmann I (2007). Effects of adenosine on functions of polymorphonuclear leukocytes from patients with septic shock. Shock.

[29] Thiel, M. *et al*. Oxygenation inhibits the physiological tissue-protecting mechanism and thereby exacerbates acute inflammatory lung injury. *PLoS Biol*. **3**, e174, 04-PLBI-RA-0506R3, 10.1371/journal.pbio.0030174 (2005). 10.1371/journal.pbio.0030174 PMC1088279 15857155

[30] Eltzschig HK, Carmeliet P (2011). Hypoxia and inflammation. N. Engl. J. Med..

[31] Nowak M (2010). The A2aR adenosine receptor controls cytokine production in iNKT cells. Eur. J. Immunol..

[32] Linden, J. Adenosine in tissue protection and tissue regeneration. *Mol*. *Pharmacol*. **67**, 1385–1387, 105.011783/10.1124/mol.105.011783 (2005). 10.1124/mol.105.011783 15703375

[33] Oppert M (2008). Acute renal failure in patients with severe sepsis and septic shock–a significant independent risk factor for mortality: results from the German Prevalence Study. Nephrol. Dial. Transplant..

[34] Anderberg SB, Luther T, Frithiof R (2017). Physiological aspects of Toll-like receptor 4 activation in sepsis-induced acute kidney injury. Acta physiol..

[35] Liu, V. X. *et al*. The Timing of Early Antibiotics and Hospital Mortality in Sepsis. *Am*. *J*. *Respir*. *Crit*. *Care Med*., 10.1164/rccm.201609-1848OC (2017). 10.1164/rccm.201609-1848OC PMC5649973 28345952

[36] Hotchkiss RS, Coopersmith CM, McDunn JE, Ferguson TA (2009). The sepsis seesaw: tilting toward immunosuppression. Nat.med..

[37] Opal SM (2010). New perspectives on immunomodulatory therapy for bacteraemia and sepsis. Int. J. Antimicrob. Agents.

[38] Huang GK, Stewardson AJ, Grayson ML (2014). Back to basics: hand hygiene and isolation. Curr. Opin. Infect. Dis..

[39] Bone RC (1987). A controlled clinical trial of high-dose methylprednisolone in the treatment of severe sepsis and septic shock. N. Engl. J. Med..

[40] Annane D (2009). Corticosteroids in the treatment of severe sepsis and septic shock in adults: a systematic review. JAMA Intern. Med..

[41] Annane, D. *et al*. Corticosteroids for treating sepsis. *Cochrane Database Syst*. *Rev*., CD002243, 10.1002/14651858.CD002243.pub3 (2015). 10.1002/14651858.CD002243.pub3 PMC6494587 26633262

[42] Heming N, Lamothe L, Ambrosi X, Annane D (2016). Emerging drugs for the treatment of sepsis. Expert Opin. Emerg. Drugs.

[43] Mathias B, Szpila BE, Moore FA, Efron PA, Moldawer LL (2015). A Review of GM-CSF Therapy in Sepsis. Medicine.

[44] Mansur A (2015). Chronic kidney disease is associated with a higher 90-day mortality than other chronic medical conditions in patients with sepsis. Sci. rep..

[45] Kelly E (2013). Comparison of arterial and venous blood biomarker levels in chronic obstructive pulmonary disease. F1000Res.

[46] Fernandez-Serrano S (2003). Molecular inflammatory responses measured in blood of patients with severe community-acquired pneumonia. Clin Diagn Lab Immunol.

[47] Shah B (2013). Comparison of platelet activity measurements by use of arterial and venous blood sampling. Journal of thrombosis and haemostasis: JTH.

[48] Sepsis, K. http://clinicaltrials.gov/show/NCT00832039.

[49] Suzuki N, Sakane T (1988). Mechanism of T cell-derived helper factor production upon stimulation with pokeweed mitogen in humans. Clin. Exp. Immunol..

[50] Waldmann TA, Broder S (1982). Polyclonal B-cell activators in the study of the regulation of immunoglobulin synthesis in the human system. Adv. Immunol..

[51] Iliodromitis EK (1998). The PKC activator PMA preconditions rabbit heart in the presence of adenosine receptor blockade: is 5′-nucleotidase important?. J. Mol. Cell. Cardiol..

[52] Nishihira J, O’Flaherty JT (1985). Phorbol myristate acetate receptors in human polymorphonuclear neutrophils. J. Immunol..

[53] Masilamani M, Kassahn D, Mikkat S, Glocker MO, Illges H (2003). B cell activation leads to shedding of complement receptor type II (CR2/CD21). Eur. J. Immunol..

[54] Touraine JL (1977). Phorbol myristate acetate: a mitogen selective for a T-lymphocyte subpopulation. J. Exp. Med..

[55] Trickett A, Kwan YL (2003). T cell stimulation and expansion using anti-CD3/CD28 beads. J. Immunol. Methods.

[56] Poltorak A (1998). Defective LPS signaling in C3H/HeJ and C57BL/10ScCr mice: mutations in Tlr4 gene. Science.

