# Supplementary material for: Early immune anergy towards recall antigens and mitogens in patients at onset of septic shock
Source: Sci Rep. 2018 Jan 29;8:1754. doi: 10.1038/s41598-018-19976-w (PMC5788998; doi:10.1038/s41598-018-19976-w)
Supplement: Supplementary file 1 — Supplemental material [file 41598_2018_19976_MOESM1_ESM.pdf]

Early immune anergy towards recall antigens and mitogens in patients at onset of septic shock

M. Feuerecker[1*], L. Sudhoff[1*], B. Crucian[2], J.-I. Pagel[1], C. Sams [2], C. Strewe[1], A. Guo[1], G. Schelling[1], J. Briegel[1], I. Kaufmann[1,3] and A. Choukèr[1]

Supplemental Material:

Study information

Inclusion Criteria

For study enrolment, patients had to meet criteria for either severe sepsis or septic shock according to the guidelines applicable at the time of this study [1]. Patients had to be enrolled within 24 hours after onset of severe sepsis or septic shock. A clinically suspected or microbiologically ascertained infection in combination with two or more of the SIRS-criteria, indicating the presence of sepsis had to be observed. In contrast to the original American College of Chest Physicians (ACCP) and the Society of Critical Care Medicine (SCCM) definitions, all interval boundaries (e.g. 38°C, 90 bpm, etc.) were included to meet the criteria. Additionally to an increased respiratory rate and a decreased $PaCO_2$, the necessity of mechanical ventilation alone qualified to fulfil the respiratory criterion. Furthermore, to fulfil the severe sepsis or septic shock definitions, one or more of the following criteria had to be present: acute encephalopathy, thrombocytopenia, renal dysfunction, metabolic acidosis, arterial hypoxemia, arterial hypotension or septic shock.

Exclusion Criteria

Patients were excluded if one or more of the following criteria existed: an infection in which guidelines suggested a long-term antimicrobial therapy, disastrous prognosis, breastfeeding period, pregnancy, selenium intoxication, severely immunologically compromised patients with $CD4^+$ counts <200/mm^3 or neutrophils <500/mm^3 or medicamentous immunosuppression, in status post solid organ transplantation, therapy limitation or termination, participation in another clinical trial within the last 30 days, current participation in another research project, earlier participation in SISPCT or a personal relation to the principal investigator.

- Relationship between white blood count and IL-1β release after LPS stimulation

WBC is an important inflammatory marker: both ends of the spectrum, leukopenia as well as leukocytosis, can indicate severe inflammatory processes and thus both conditions represent one of the four SIRS criteria [1]. We hypothesized that the WBC might be correlated with cytokine release. After calculating Spearman's rank correlation coefficients, significant correlations on the 0.95 significance level could only be found in three out of 30 tested read-outs in a two-tailed test. In two out of the three cases, a negative correlation was found, while the remaining case showed a positive correlation indicating that no stringent linear correlation is present (Suppl. Figure 1).

Suppl. Figure 1: Relationship between white blood count and IL-1β release after stimulation with LPS

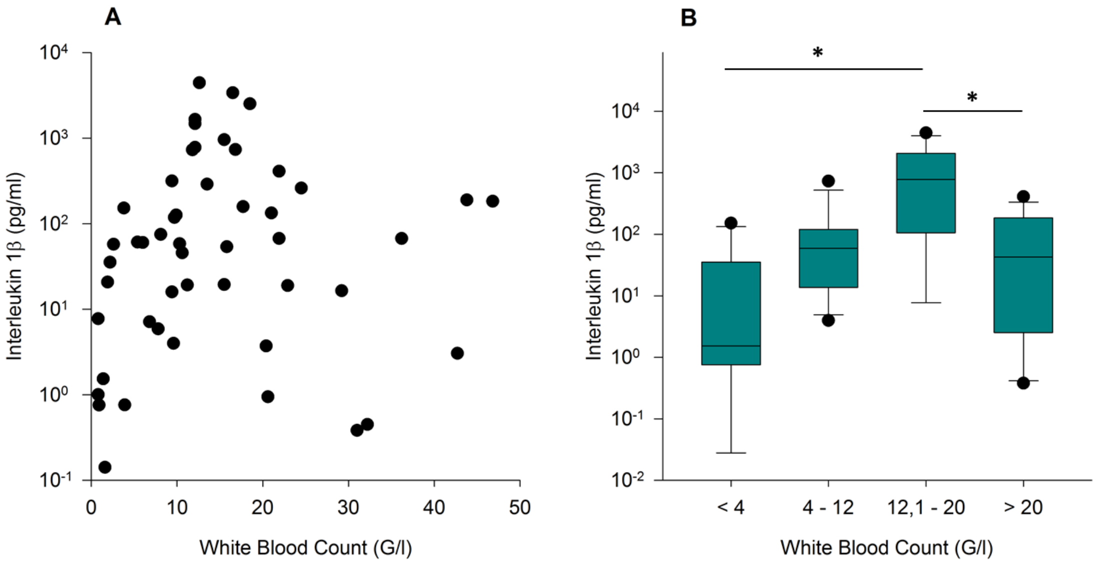

Suppl. Figure 1 **A** shows a scatterplot for patients' WBC versus IL-1β release after stimulation with LPS. It suggests a non-linear correlation with leukopenic and severely leukocytotic patients showing the faintest IL-1β release. Suppl. Figure 1 **B** shows the corresponding boxplot after allocation of the patients to four groups (**I)** leukopenia (WBC < 4G/l), **II)** normal WBC (4-12G/l), **III)** moderate leukocytosis (WBC 12,1-20G/l) and **IV)** severe leukocytosis (WBC>20G/l)). Statistically significant differences are marked * $p < 0.05$ (One-way ANOVA on RANKS followed by Dunn´s test).

- Relationship between serum IL-6 and IL-1β release after LPS stimulation

IL-6 serves as a reliable inflammatory marker in modern critical care medicine, and is characterized by a fast dynamic with a biological half-life of less than 6 hours [2]. It thus represents an early indicator of inflammatory processes.

Results evidenced a negative correlation of baseline IL-6 serum of septic shock patients versus IL-1β release after stimulation with LPS (Suppl. Figure 2 A). To further statistically assess the underlying correlation, quartile groups were formed for IL-6 baseline values (Suppl. Figure 2B), proving the statistical significance of the above mentioned negative correlation. This correlation can be seen as a blood marker of the disease severity.

Suppl. Figure 2: Relationship between IL-6 and IL-1β release after stimulation with LPS

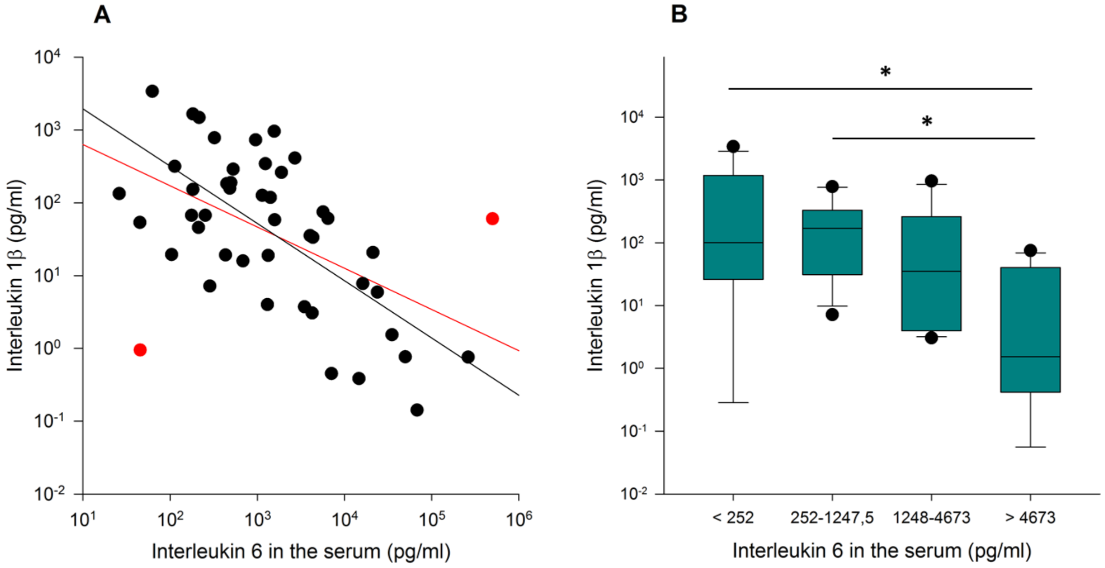

X-axis represents the serum IL-6 level, y-axis shows the IL-1β release from whole blood after stimulation with LPS. **A** Scatterplot with regression line (*red line*). Two outliers are highlighted in red. The *black line* represents the resulting regression line without the outliers. Axis scale: logarithmic. **B** Patients were allocated to quartile groups depending on their IL-6 measured in the blood serum. The outliers identified in A) were included into calculation. Statistically significant differences are indicated *p<0.05 (One-way ANOVA on RANKS followed by Dunn´s test).

- *Mortality*

Mortality in the studied patients was 20 % (14 deaths) during the 90 days period (7 patients could not entirely be followed up). By employing Receiver operating characteristic (ROC) curves, the release of IFN-γ after stimulation with PWM allowed a statistically significant prediction of overall death risk, with lower IFN-γ release indicating higher mortality (Area under the receiver operating characteristic curve (AUC) = 0.72, p = 0.01). The other read-outs, particularly our reference read-outs PWM - TNF-α and LPS - IL-1β, did not show any significant values in the ROC-analysis regarding mortality (Suppl. Table 1, Suppl. Figure 3).

Compared to this, the disease severity classification systems SAPS II and APACHE II also did not reveal statistically significant predictability of mortality. Altogether characterized by p-values > 0.05, AUCs were 0.61 for SAPS II Baseline and 0.64 for APACHE II Baseline (Suppl. Table 1, Suppl. Figure 3).

When incorporating all of the parameters (PWM – TNF-α, LPS – IL-1β, serum IL-6, APACHE II, SAPS II) by mathematically combining them in a single ROC curve, however, a result scarcely significant on the 0.05 significance level results.

Suppl. Table 1: Performance of different markers in ROC-analysis regarding 90-day mortality

Marker	N	AUC (95 % CI)	p	Cut off	Sensitivity	Specificity
PWM - TNF-α	75	0.59 (0.44 – 0.75)	> 0.05	< 29.4 pg/ml	79 %	44 %
LPS - IL-1β	56	0.62 (0.43 – 0.81)	> 0.05	< 40.5 pg/ml	67 %	64 %
SAPS II	73	0.61 (0.42 – 0.79)	> 0.05	> 82	36 %	90 %
APACHE II	73	0.64 (0.49 – 0.79)	> 0.05	> 25.5	86 %	44 %
Serum IL-6	61	0.67 (0.51 – 0.84)	> 0.05	> 1195 pg/ml	77 %	56 %
PWM - TNF-α + APACHE II + serum IL-6	61	0.63 (0.47 – 0.78)	> 0.05	Fictious value	92 %	42 %
All of the above combined	47	0.72 (0.54 – 0.89)	< 0.05	Fictious value	75 %	60 %

ROC: Receiver operating characteristic, AUC: Area under the receiver operating characteristic curve, CI: Confidence interval.

Suppl. Figure 3: ROC curves for the endpoint 90-day mortality

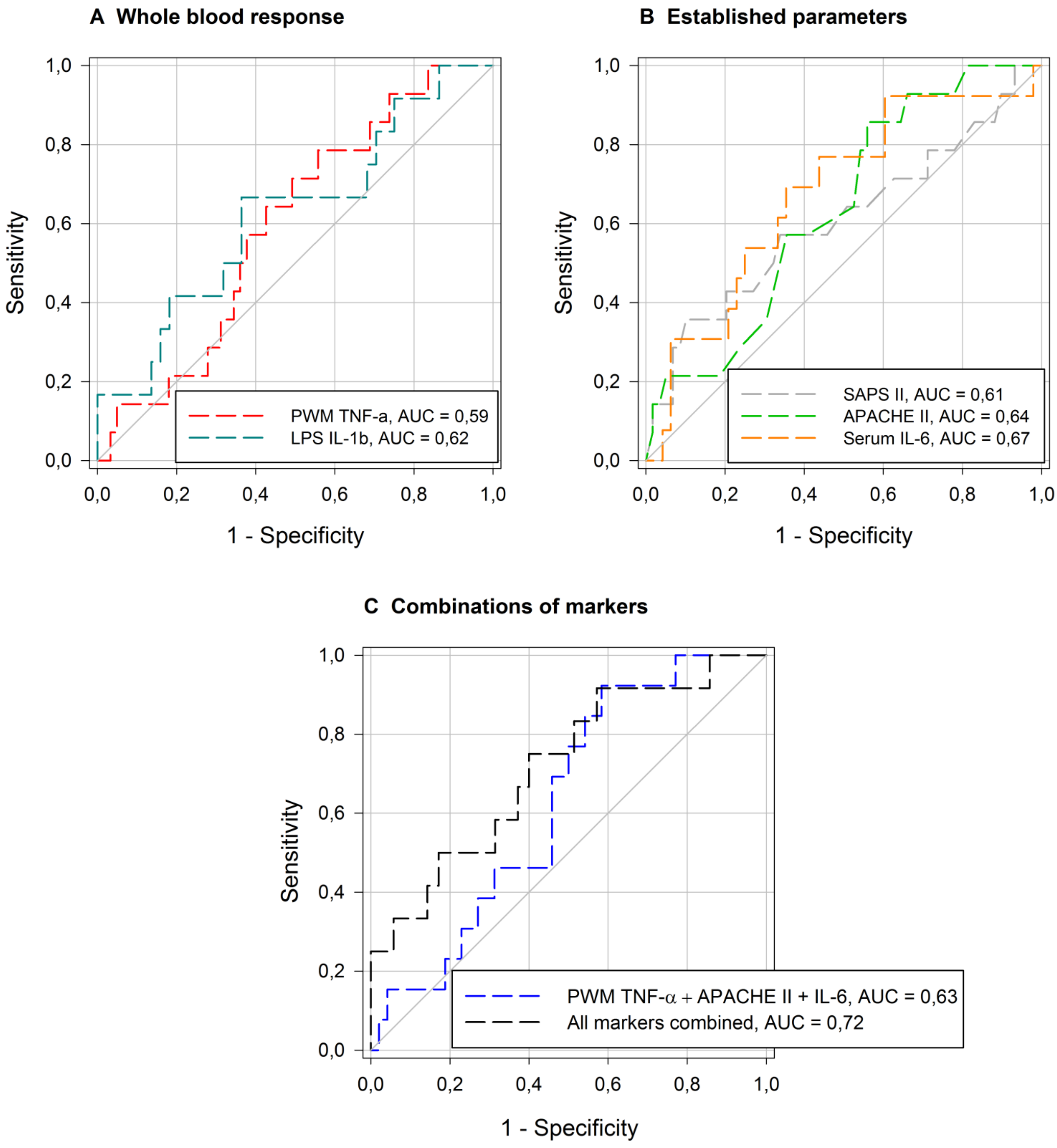

ROC-curves for A) whole blood response, B) established parameters used in sepsis and C) different combinations of markers referred to in A) and B), regarding the endpoint 90-day mortality. ROC: Receiver operating characteristic, PWM: Pokeweed mitogen, TNF: Tumor necrosis factor, AUC: Area under the receiver operating characteristic curve, LPS: Lipopolysaccharide, IL: Interleukin, SAPS II: Simplified Acute Physiology Score, APACHE II: Acute Physiology and Chronic Health Evaluation II, All markers combined: combination of PWM TNF-α, LPS IL-1β, SAPS II, APACHE II and serum IL-6.

References

1 American College of Chest Physicians/Society of Critical Care Medicine Consensus Conference: definitions for sepsis and organ failure and guidelines for the use of innovative therapies in sepsis. *Crit. Care Med.* **20**, 864-874 (1992).

2 Ridker, P. M., Rifai, N., Stampfer, M. J. & Hennekens, C. H. Plasma concentration of interleukin-6 and the risk of future myocardial infarction among apparently healthy men. *Circulation* **101**, 1767-1772 (2000).
